# Sex-Related Differences in Pulmonary Function following 6 Months of Cigarette Exposure: Implications for Sexual Dimorphism in Mild COPD

**DOI:** 10.1371/journal.pone.0164835

**Published:** 2016-10-27

**Authors:** Anthony Tam, Jason H. T. Bates, Andrew Churg, Joanne L. Wright, S. F. Paul Man, Don D. Sin

**Affiliations:** 1 Department of Medicine, Centre for Heart Lung Innovation (St. Paul's Hospital), Vancouver, British Columbia, Canada; 2 Department of Medicine, University of Vermont College of Medicine, Burlington, Vermont, United States of America; 3 Department of Pathology, University of British Columbia, Vancouver, British Columbia, Canada; Forschungszentrum Borstel Leibniz-Zentrum fur Medizin und Biowissenschaften, GERMANY

## Abstract

Female smokers have increased risk of chronic obstructive pulmonary disease (COPD) compared with male smokers who have a similar history of cigarette smoke exposure. We have shown previously that chronic smoke exposure for 6 months leads to increased airway wall remodeling in female C57BL/6 mice compared with male C57BL/6 mice. These differences, however, were not evident in female ovariectomized mice exposed to cigarette smoke. Herein, we report on the pulmonary function test results from the flexiVent system, which was used to determine the potential functional consequences of the histologic changes observed in these mice. We found that tissue damping (*G*) was increased in female compared to male or ovariectomized female mice after smoke exposure. At low oscillating frequencies, complex input resistance (*Zrs*) and impedance (*Xrs*) of the respiratory system was increased and decreased, respectively, in female but not in male or ovariectomized female mice after smoke exposure. Quasistatic pressure-volume curves revealed a reduction in inspiratory capacity in female mice but not in male or ovariectomized female mice after smoke exposure. The remaining lung function measurements including quasistatic compliance were similar amongst all groups. This is the first study characterizing a sexual dimorphism in respiratory functional properties in a mouse model of COPD. These findings demonstrate that increased airway remodeling in female mice following chronic smoke exposure is associated with increased tissue resistance in the peripheral airways. These data may explain the importance of female sex hormones and the increased risk of airway disease in female smokers.

## Introduction

Chronic obstructive pulmonary disease (COPD) is a complex disease that includes both emphysema and airway remodelling, but these pathologic changes may be influenced by female sex hormones. In patients with very severe COPD, women have anatomically smaller lumens and thicker walls of airways less than 2 mm in diameter compared to men [1]. Epidemiological studies have also shown that female smokers experience a more rapid loss in lung function compared with male smokers who smoke a similar number of cigarettes per day [2]. Van Winkle and colleagues have demonstrated that female mice demonstrated greater proximal airway injury than male mice after acute injection of napthalene, an important product in side-stream cigarette smoke [3]. Female A/J mice develop emphysema sooner than male A/J mice after 10 weeks and 16 weeks of cigarette smoke exposure, respectively [4]. Collectively, these data have demonstrated important biological sex differences in emphysema and airways disease.

In a recent study, we showed that chronic smoke exposure was associated with increased histological changes of small airway remodelling in female compared with male or female ovariectomized mice even though there were no significant sex-related differences in the histological burden of emphysema [5]. These findings were accompanied by corresponding increases in the real part of respiratory system impedance, *Zrs*, in the female mice. This suggested the existence of a definable link between lung function and lung structure that may be modified by smoking and sex hormones. To further characterize the functional consequences of sex hormones on the airways and the lung parenchyma of mice exposed to long term cigarette smoke, we performed extensive physiological measurements in both male and female (ovariectomized as well as ovary intact) mice; the findings of these experiments have not been reported in detail previously [5]. These data are important because symptoms, quality of life and prognosis are determined to a large extent by lung function (as opposed to lung architecture) in human patients. In this study, we determined the effects of long term cigarette smoke exposure on lung function and volumes in male mice and in female mice with and without their ovaries.

## Materials and Methods

### Experimental approach

The data reported in the present study were collected from the same mice at the same time as the data reported in a previous publication from our laboratory. This previous publication dealt primarily with the morphological, molecular, and biochemical changes resulting from chronic cigarette exposure in the setting of sex hormones (6). In the present study we report additional data collected during the study that allowed us to construct a more detailed link between structure and function in COPD in the setting of sex hormones.

### Animals

As described previously [5], adult male, female and ovariectomized C57BL/6 mice (12 weeks old) were obtained from Charles River (Montreal, PQ, Canada). Surgical ovariectomy of female mice was performed at Charles River four weeks prior to cigarette smoke exposure. 1R1 and 2R4F research grade cigarettes were obtained from the University of Kentucky (Lexington, KY).

### Smoke exposure

As described previously [5], we studied 6 groups of mice (n = 10 per group): 1) male control, 2) male smoke-exposed, 3) female control, 4) female smoked-exposed, 5) ovariectomized control, and 6) ovariectomized smoke-exposed. The smoke-exposed groups were exposed to three cigarettes (one 1R1 and two 2R4F with the filters removed, or two 1R1 and one 2R4F with the filters removed on every other smoking day) for 5 days per week for 6 months. The filters were removed from the cigarettes in order to increase the effective dose of smoke delivered to the mice and to accelerate development of emphysema in our C57BL/6 mice. All smoke exposures were conducted using our standard nose-only smoke exposure system. Our system described herein has been previously shown to produce a carboxyhemoglobin level of ~5% in mice [6], a value that is similar to that of human smokers who smoke 10 cigarettes per day [7].

### Pulmonary function assessment

As described previously [5], immediately after the last smoke exposure, all mice except 2 smoke-exposed ovariectomized mice from the 6 month study indicated above were anesthetized (150mg/kg ketamine and 10mg/kg xylazine), tracheostomized (closed thorax) with an 18-gauge blunted needle advanced 5 tracheal rings and secured with silk ties. An additional dose of a ketamine/xylazine mix (25% of initial dose) was given 30 min from the initial injection. The tracheal cannula was connected to a computer-controlled small animal ventilator (flexiVent; SCIREQ, Montreal, Canada). Mice were mechanically ventilated in a supine position at a respiratory rate of 150 breaths/min and at a tidal volume of 10ml/kg, with a pressure limit of 30 cmH_2_O coupled to a constant positive expiratory end-pressure (PEEP) of 3 cmH_2_O. Muscle paralysis was achieved using pancuronium (2mg/kg intraperitoneally) to prevent respiratory efforts during the measurement. A 60 W incandescent light bulb was placed 30 cm directly above the mouse to maintain body temperature. Heart rate was monitored with an electrocardiogram (ECG) (SCIREQ, Montreal, Canada) attached to the limbs of the animal to check for proper anesthetic depth. Before each mouse was connected to the flexiVent, we collected calibration signals by applying a volume perturbation initially through a completely closed tracheal cannula and then opened to the atmosphere to estimate the flow resistance of the tracheal cannula and the elastance of air in the ventilator cylinder. To provide a constant volume history, a 6 second deep inflation maneuver to 27 cmH_2_O was performed twice prior to data collection. An 8s volume perturbation signal containing frequencies between 1 and 20.5 Hz (primewave-8, SCIREQ, Montreal, Canada) with a peak-peak amplitude of 3 ml/kg was then applied to the lungs to measure the mechanical impedance of the respiratory resistance (*Zrs*) and impedance (*Xrs*). Fitting the constant-phase model of *Zrs* provided estimates of airway resistance (*Rn*), tissue damping (*G*) and tissue elastance (*H*) [8]. Model fits were accepted only if the coefficient of determination of the fit was > 0.95. Three measurements of *Zrs* were obtained under each experimental condition and the resulting values of *Rn*, *G* and *H* were averaged. Pressure-volume (PV) loops were generated by applying a series of volume-steps that inflated to the lungs to 30 cmH_2_O followed by deflation, all in the complete absence of spontaneous breathing efforts. Fitting the Salazar-Knowles equation to the expiratory limbs of the PV loops provided a measure of the exponent *K* that reflects the curvature of the PV relationship [9].

### Measurement of airway responsiveness

Frequency-dependent respiratory resistance (*Zrs*) and reactance (*Xrs*) curves were also generated from all groups of mice using initially nebulization of phosphate-buffered saline (PBS) as the vehicle, followed by increasing doses of methacholine of 1.6, 3.1, 6.25, 12.5 and 25mg/ml (A2251, Sigma Aldrich) over 10s during the inspiratory phase of the ventilation pattern at a constant flow rate of 0.05ml/s. Measurements were made over a series of 10 consecutive days. In order to avoid time effects in our data, we measured all lung function parameters in one mouse from each of the 6 groups on each of the 10 days. Measurements on a control mouse and its smoked counterpart (male, female, or ovariectomized groups) were performed immediately one after the other. At the end of the experiment, mice were removed from the mechanical ventilator and were sacrificed by exsanguination.

### Statistics

Data were analyzed using parametric (normal distribution) or non-parametric (non-normal distribution) t-test, A two-way ANOVA with Bonferroni`s multiple comparisons test comparing the mean between sex-matched control vs. smoke-exposed groups was performed at each oscillation frequency, and linear regression was used to compare continuous variables. All data were analyzed using GraphPad Prism version 6 (GraphPad Software Inc, San Diego, CA, USA) and are expressed as mean ± SEM. Statistical significance was considered at P < 0.05.

### Study approval

All procedures were approved by the University of British Columbia Animal Care Committee (A11-0149).

## Results

### Lung mechanics of mice after chronic smoke exposure

**Fig 1:**
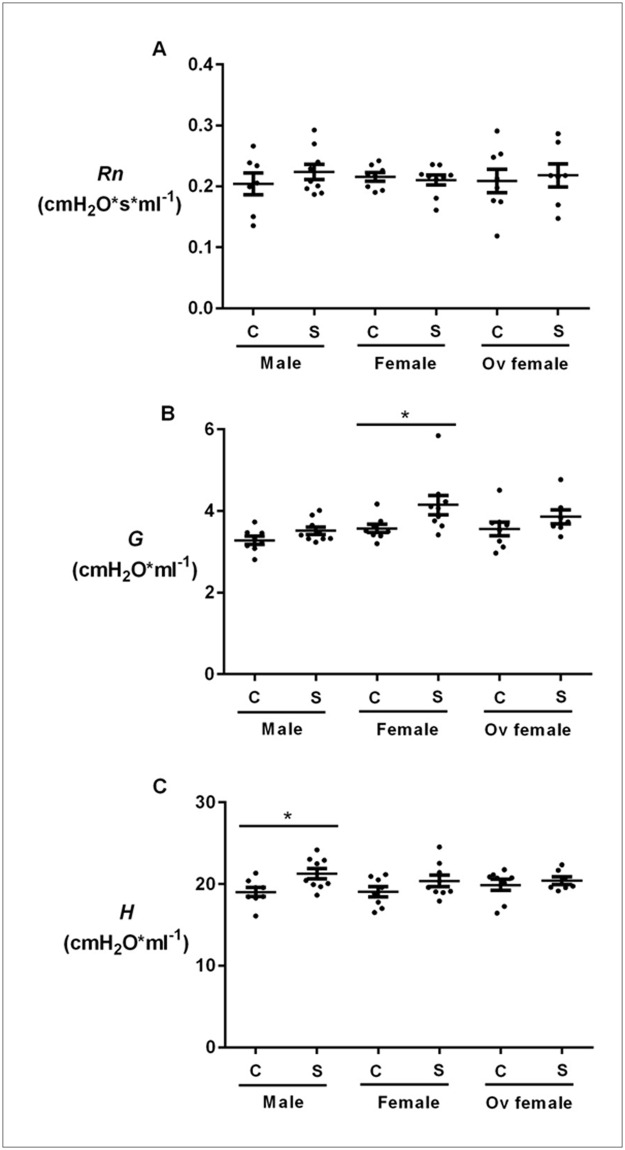
The effects of chronic smoke exposure on airway resistance, tissue damping and tissue elastance in male, female and ovariectomized mice. Female mice exposed to cigarette smoke demonstrated greater tissue damping (*G*) than control female mice. These differences were not present in male or ovariectomized female mice. Cigarette exposure significantly increased tissue elastance (*H*) in male but not female mice. A) Airway resistance (*Rn*), B) tissue damping (*G*) and C) tissue elastance (*H*) were measured in control (C) and smoke-exposed (S) male, female and ovariectomized mice. Values are expressed as mean ± SEM from N = 7–9 per group. *p<0.05 represents statistical significance. Non-parametric t-test was used in panel B, and parametric t-test was used panel C.

Cigarette smoke exposure was not associated with any significant increases in Newtonian resistance (*Rn*) in male, female (with intact ovaries) or ovariectomized mice (Fig 1A). However, smoke exposure significantly increased tissue resistance (*G*) in female but not in male mice, or ovariectomized female mice (Fig 1B). Smoke-exposed female mice demonstrated significantly greater *G* than smoke-exposed male mice (S1 Fig). In contrast, male mice exposed to cigarette smoke experienced a significant increase in tissue elastance (*H*) (Fig 1C). However, no significance difference in *H* was found between smoke-exposed male and female mice (S1 Fig). The findings were similar to the frequency-dependent respiratory resistance (*Zrs*) measurements as shown in Table 1. Specifically, cigarette smoke exposure increased *Zrs* at the two lowest frequencies (1.0 and 1.5Hz) in female mice but not in male or ovariectomized female mice. Similarly, cigarette exposure decreased frequency-dependent respiratory reactance (*Xrs*) at the two lowest frequencies in female but not male or ovariectomized mice (Table 2). We have previously demonstrated in air-exposed animals that female mice have increased peripheral airway resistance compared to male mice [5], suggesting that the distal airways of female mice may be intrinsically narrower than those of male mice. Methacholine challenge was used as positive controls in all groups of mice to demonstrate that chronic smoke exposure was not associated with smooth muscle-dependent increase in *Zrs* (S2 Fig).

**Table 1:** Effects of chronic smoke exposure on frequency-dependent respiratory resistance (Zrs) in male, female and ovariectomized mice.

	Male mice		Female mice		Ovariectomized mice	
Hz	Control	Smoked	Control	Smoked	Control	Smoked
1.0	1.0±0.08	0.9±0.02	1.0±0.02	1.1±0.05***	0.9±0.03	1.0±0.04
1.5	0.7±0.06	0.7±0.01	0.7±0.02	0.8±0.04*	0.6±0.03	0.7±0.04
2.5	0.6±0.05	0.5±0.01	0.6±0.02	0.6±0.03	0.5±0.02	0.6±0.02
8.5	0.4±0.05	0.3±0.03	0.3±0.01	0.3±0.01	0.3±0.02	0.3±0.02
20.5	0.3±0.04	0.3±0.01	0.3±0.01	0.3±0.01	0.3±0.02	0.3±0.01

Hz = oscillating frequency in Hertz. Values are expressed as mean ± SEM with N = 7–9 per group. A two-way ANOVA with Bonferroni`s multiple comparisons test comparing the mean *Zrs* between sex-matched control and smoke-exposed groups was used at each of the indicated oscillation frequencies. Statistical significance was achieved at 1.0 and 1.5Hz in female mice.

***p<0.001,

*p<0.05 compared between control and smoke-exposed female mice.

**Table 2:** Effects of chronic smoke exposure on frequency-dependent respiratory reactance (*Xrs*) in male, female and ovariectomized mice.

	Male mice		Female mice		Ovariectomized mice	
Hz	Control	Smoked	Control	Smoked	Control	Smoked
1.0	-3.8±0.3	-4.1±0.1	-3.7±0.1	-4.1±0.1****	-3.7±0.1	-4.0±0.1
1.5	-2.7±0.2	-2.9±0.09	-2.6±0.1	-2.9±0.1***	-2.6±0.1	-2.8±0.1
2.5	-1.6±0.1	-1.8±0.05	-1.6±0.05	-1.8±0.07	-1.6±0.06	-1.8±0.05
8.5	-0.7±0.07	-0.7±0.03	-0.6±0.02	-0.7±0.03	-0.6±0.03	-0.7±0.03
20.5	-0.3±0.02	-0.3±0.01	-0.3±0.02	-0.3±0.02	-0.3±0.01	-0.3±0.02

Hz = oscillating frequency in Hertz. Values are expressed as mean ± SEM with N = 7–9 per group. A two-way ANOVA with Bonferroni`s multiple comparisons test comparing the mean *Xrs* between sex-matched control and smoke-exposed groups was used at each of the indicated oscillation frequencies. Statistical significance was achieved at 1.0 and 1.5Hz in female mice.

****p<0.0001,

***p<0.001 compared between control and smoke-exposed female mice.

**Fig 2:**
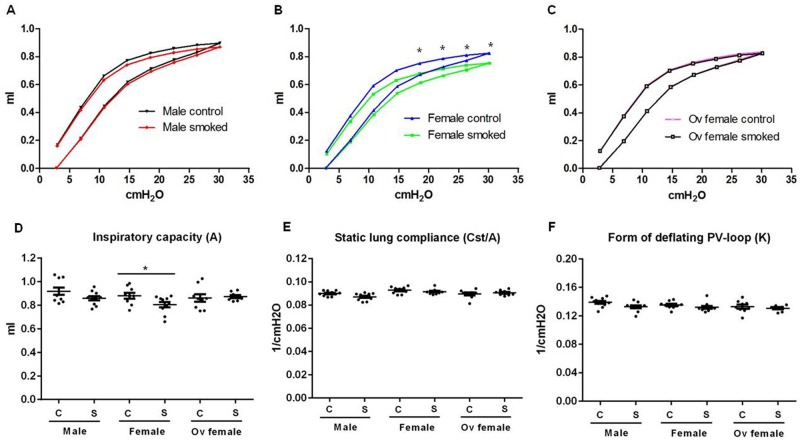
Pressure-curve loops and inspiratory capacity measurements following 6 months of cigarette exposure in male, female and ovariectomized female mice. Female mice have lower inspiratory capacity than male mice after smoke exposure, and this effect was abolished after ovariectomy. Pressure-volume (PV) loops were shown in control and smoke-exposed A) male, B) female, and C) ovariectomized mice. D) Inspiratory capacity (A), E) quasi-static lung compliance with normalization to A, and F) the form of the deflating PV-loop (*K*) are shown. Values are expressed as mean ± SEM with N = 8–10 per group. A two-way ANOVA with Bonferroni`s multiple comparisons test comparing the mean inspiratory volume between sex-matched control and smoke-exposed groups was used at each pressure step in Fig 2A-C. Statistical significance *P<0.05 was achieved at the upper 4 pressure steps of the deflation PV-loops in female mice. Parametric t-tests were used in panels D-F.

Cigarette smoke exposure shifted the inspiratory and expiratory PV curve in a rightward direction in female but not in male or ovariectomized mice (Fig 2A–2C). Similarly, with cigarette exposure, female mice experienced a significant reduction in the inspiratory capacity (*A*), determined from the fit of the Salazar-Knowles equation to the expiratory limb of the PV loop (Fig 2D). Quasi-static lung compliance (*Cst*) was, however, not significantly different between the groups after normalization to the inspiratory capacity within each group (Fig 2E). The curvature of the deflation limb of the PV-loop (*K*) was not significantly different between all groups (Fig 2F).

### Body weight

**Fig 3:**
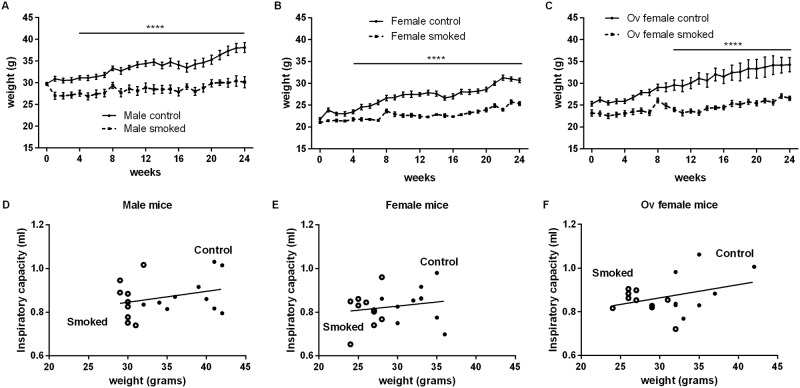
Inspiratory capacity was not related to the body weight of mice. Total body weight of control (solid line) and smoke-exposed (dashed line) A) male, B) female, and C) ovariectomized mice was measured weekly over 24 weeks of smoke exposure. Values are expressed as mean ± SEM with N = 8–10 per group. A two-way ANOVA with Bonferroni`s multiple comparisons test comparing the mean total body weight between sex-matched control and smoke-exposed groups was used at each week for a total of 24 weeks of exposure in Fig 3A-C. Statistical significance of ****P<0.0001 was achieved at all levels from week 4–24 of exposure in both male and female mice, and from week 10–24 of exposure in ovariectomized mice. The relationship between inspiratory capacity and total body weight of control (open circle) and smoke-exposed (dark circle) D) male, E) female and F) ovariectomized mice are shown. Linear regression analyses were used in panels D-F with N = 7–9 per group.

Body weight increased by 21%, 33% and 29% in control male, female and ovariectomized mice, respectively, from 3 to 9 months of age (Fig 3A–3C). However, chronic smoke exposure reduced the ability to gain weight in all groups of mice by decreasing body weight by 20–25%. To determine whether differences in body weight influenced inspiratory capacity, we plotted inspiratory capacity versus body weight but found no significant relationships (Fig 3D–3F). Furthermore, there was no significant difference in total body weight changes (sex-matched control vs. smoke-exposed mice) over the experimental period between female and ovariectomized mice (S3 Fig).

## Discussion

In patients with severe COPD, female smokers are at increased risk of small airways disease compared with male smokers [1]. In mild to moderate COPD, female smokers have an accelerated decline in lung function compared with male smokers with similar pack-years of smoking exposure [10]. However, because there are significant differences in the amount and manner of smoking between men and women [11], the influence of confounding factors could not be fully resolved in these observational studies.

To our knowledge, the present study is the first of its kind to comprehensively evaluate sex-related differences in lung function following 6 months of cigarette exposure in mice with and without intact ovaries. We found that 6 months of cigarette exposure, which produces mild histologic changes of emphysema and small airway remodelling in these mice that mimic mild COPD in humans [12,13], resulted in significant perturbations in lung function reflective of disease in the “small airways” of female mice but not in male or ovariectomized mice.

We found the greatest impact on lung function related to impedance parameters tissue resistance (*G*) and tissue elastance (*H*). Lung tissues exhibit a resistance to motion in the same way that air exhibits a resistance to motion through an airway. Thus, in a perfectly homogeneous lung, *G* indicates the extent to which energy is dissipated in the respiratory tissue as lung volume increases and decreases. Similarly, *H* is a measure of lung elastance of the tissues that opposes expansion as lung volume increases. The fact that *G* and *H* are both non-zero attests to the viscoelastic nature of the tissues. However, *G* and *H* may also be increased by derecruitment of airspaces, by an increase in the regional heterogeneity of lung mechanics, or by a reduction in lung size. Smoking did not significantly affect lung size (Fig 3D–3F), but we found that smoking increased *G* in the female group (Fig 1B) and *H* in the male group (Fig 1C). The increased *G* may reflect functional consequences of increased airway remodelling in the females relative to the males and ovariectomized female mice in response to cigarette smoke as in these mice we observed increased collagen deposition in the small airways as previously described [5]. We thus posit that females with intact ovaries experienced an increase in the intrinsic resistive properties of their lung tissues and/or increased regional lung heterogeneity related to chronic smoke exposure compared with male or ovariectomized female mice.

We also found that cigarette exposure elevated resistance and decreased reactance at the two lowest frequencies measured only in the female mice (Tables 1 and 2). Resistance and reactance at low frequencies determine *G* and *H*, respectively, and were generated using fits provided by the constant-phase model [8]. However, although these model fits are generally extremely good, they are never perfect because the constant-phase model does not perfectly describe actual impedance. These errors in fit may thus explain our otherwise curious observation of an increase in *H* only in the male mice (Fig 1C). We postulate that because the effects of smoking on impedance are in general very subtle, even small errors in model fitting could lead to spurious results. This is different from the situation in human patients with COPD who typically show much greater effects on mechanics due to smoking than those observed in mice. Despite these important differences, it has been shown previously that patients with COPD exhibit increased *G*, *H*, and hysteresivity, indicative of pathology in the small, peripheral airways [14], similar to what we observed in mice.

Interestingly, COPD caused by cigarette smoking is often associated with dynamic hyperinflation and increased lung compliance, which would be expected to a decrease *H* and increase inspiratory capacity [15]. We previously showed that smoke exposure produced comparable (and mild) degrees of emphysema in the parenchyma of male, female, and ovariectomized female mice [5], but inspiratory capacity was decreased in the smoke-exposed female mice while remaining unchanged in the other two groups (Fig 2). One possibility is that residual volume may have increased in the female mice after chronic smoke exposure, corresponding to dynamic hyperinflation in patients with emphysema, which may reflect an increased tendency for remodelled peripheral airways to collapse at low lung volumes. In support of our observations, Sasaki and colleagues showed that female C57BL/6 mice have increased end-expiratory lung volume after 20 weeks of cigarette smoke exposure [16]. Furthermore, total lung capacity was increased in mice exposed to 16 weeks of smoke exposure compared to wild type mice [17]. On the other hand, it is by no means certain that the pathology of smoke exposure in mice will precisely recapitulate that seen in humans. Mice have much larger airways relative to lung size and fewer airway generations compared with human lungs [18]. Thus, cigarette exposure-related airway remodelling may have differential effects on overall lung mechanics between mice and humans.

However, we must also acknowledge the limitations imposed on our ability to draw conclusions by the subtlety of the histologic changes of airway remodelling and emphysema produced by smoke exposure [5]. The histologic changes were modest, which may explain the relatively small functional changes that were observed in this study (Figs 1–3). Nevertheless, our data consistently demonstrated an increase in G (Fig 1B and S1A Fig) and a decrease in inspiratory capacity (Fig 2D) that were more pronounced in female than male mice after chronic smoke exposure, suggesting a differential response related to sex. We have previously demonstrated a female sex-hormone-associated increases in oxidative stress and peripheral airway wall thickening in response to chronic smoke exposure. We also showed that even in control non-smoked mice (exposed to ambient air), females had increased peripheral airway resistance compare with males, which worsened with cigarette smoke exposure [5]. Together, these data suggested that there are important sex-related differences in lung structure and function that are exaggerated with cigarette smoke exposure.

In summary, we have found significant changes in lung mechanics in smoke-exposed female but not male mice, which were abolished by ovariectomy, implicating a key role for sex hormones in the pathogenesis of smoking-related COPD. Six months of smoking increased *Zrs* and decreased *Xrs* in the peripheral airways and caused a reduction in the inspiratory capacity in female but not male mice. These results provide experimental data to support the concept of increased susceptibility of females towards COPD with similar cigarette exposure, which associates with not only histologic changes but also functional changes in both the parenchyma and peripheral airways.

## Supporting Information

S1 Fig Tissue damping and elastance between smoke-exposed male and female mice. A) Tissue damping (*G*) and B) tissue elastance (*H*) were compared between smoke-exposed male and female mice. Values are expressed as mean ± SEM from N = 9 per group. ** p<0.01 represents statistical significance. Non-parametric t-test was used in panel A, and parametric t-test was used panel B.
(TIF)

S2 Fig Dose-response of respiratory resistance and impedance to methacholine challenge. Frequency-dependent respiratory resistance (Zrs) obtained from air-exposed and smoke-exposed male (A-B), female (C-D) and ovariectomized (E-F) mice nebulized with PBS as vehicle or 1.6, 3.1, 6.25, 12.5, 25mg/ml of methacholine are shown. Values are expressed as mean ± SEM with 7–9 mice per group. A two-way ANOVA with Bonferroni`s multiple comparisons test comparing the mean *Zrs* in each group of mice were performed in the indicated panels at each of the oscillation frequencies. Statistical significance of at least *P<0.05 was achieved at all oscillation frequencies when compared between PBS vehicle control and 25mg/ml of methacholine in all groups of mice.
(TIF)

S3 Fig Change in weight between female (ovary-intact) and ovariectomized mice. The change in whole body weight between control (air) and smoke-exposed mice were compared between female (ovary-intact) and ovariectomized mice over 24 weeks of exposure. Values are expressed as mean ± SEM with N = 8–10 per group. A two-way ANOVA with Bonferroni`s multiple comparisons test comparing the mean change in weight between air and smoke exposure in ovariectomized (Ov) and non-ovariectomized mice was performed. No statistical significance was achieved at any of the time points.
(TIF)
