# Supplementary figures and images for: Sex-Related Differences in Pulmonary Function following 6 Months of Cigarette Exposure: Implications for Sexual Dimorphism in Mild COPD
Source: PLoS One. 2016 Oct 27;11(10):e0164835. doi: 10.1371/journal.pone.0164835 (PMC5082824; doi:10.1371/journal.pone.0164835)

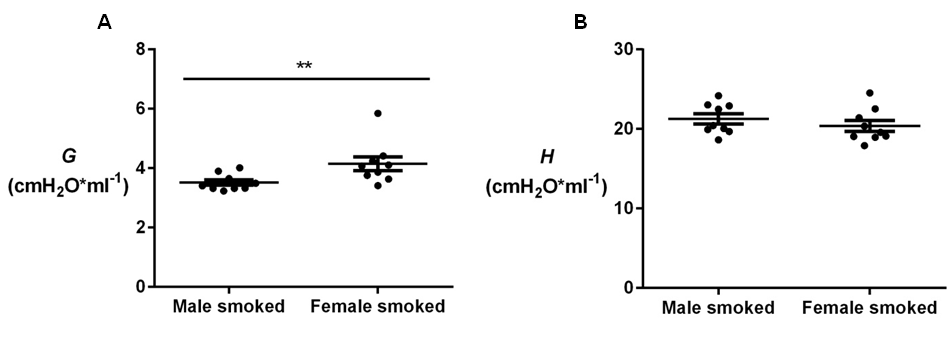

Supplement: S1 Fig — A) Tissue damping (G) and B) tissue elastance (H) were compared between smoke-exposed male and female mice. Values are expressed as mean ± SEM from N = 9 per group. ** p<0.01 represents statistical significance. Non-parametric t-test was used in panel A, and parametric t-test was used panel B. (TIF) [file pone.0164835.s001.tif]

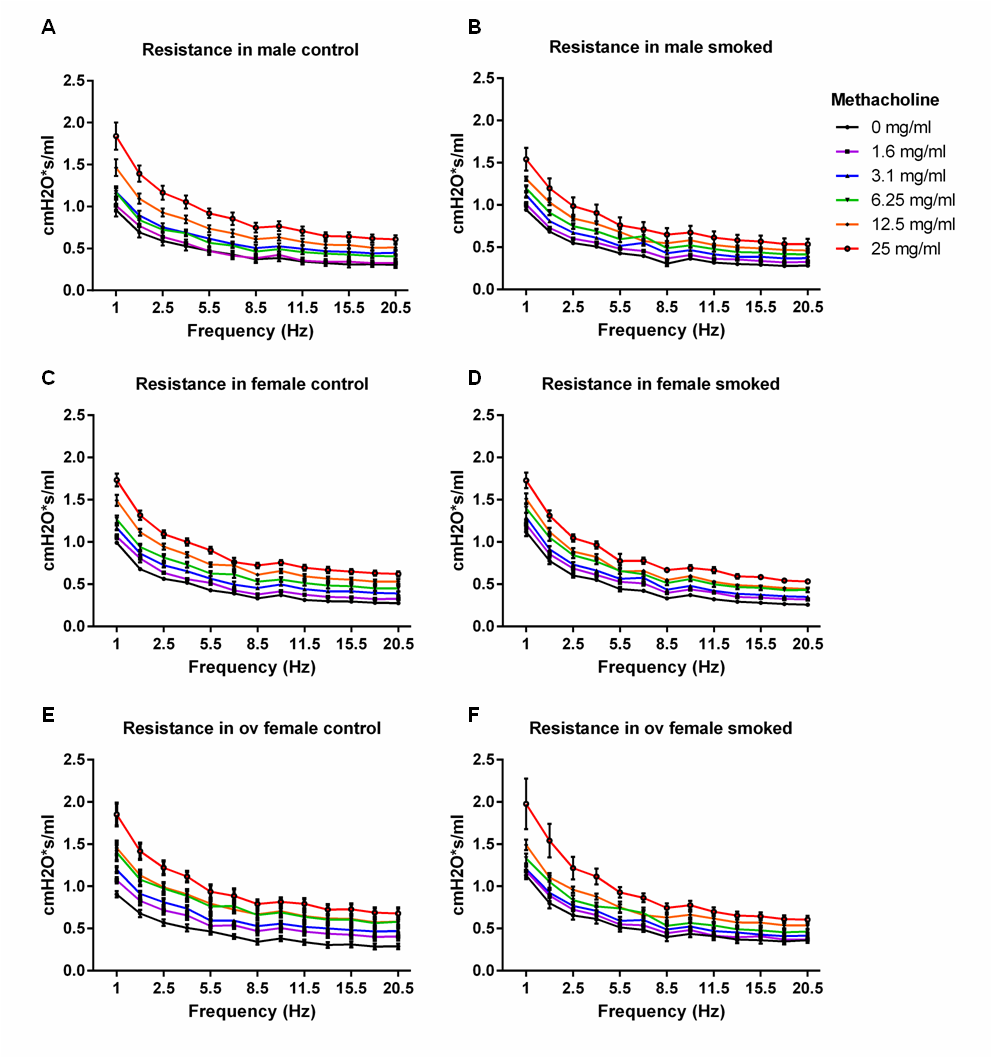

Supplement: S2 Fig — Frequency-dependent respiratory resistance (Zrs) obtained from air-exposed and smoke-exposed male (A-B), female (C-D) and ovariectomized (E-F) mice nebulized with PBS as vehicle or 1.6, 3.1, 6.25, 12.5, 25mg/ml of methacholine are shown. Values are expressed as mean ± SEM with 7–9 mice per group. A two-way ANOVA with Bonferroni`s multiple comparisons test comparing the mean Zrs in each group of mice were performed in the indicated panels at each of the oscillation frequencies. Statistical significance of at least *P<0.05 was achieved at all oscillation frequencies when compared between PBS vehicle control and 25mg/ml of methacholine in all groups of mice. (TIF) [file pone.0164835.s002.tif]

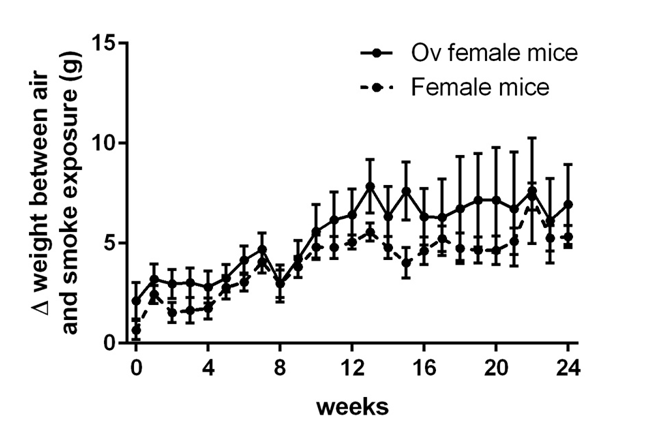

Supplement: S3 Fig — The change in whole body weight between control (air) and smoke-exposed mice were compared between female (ovary-intact) and ovariectomized mice over 24 weeks of exposure. Values are expressed as mean ± SEM with N = 8–10 per group. A two-way ANOVA with Bonferroni`s multiple comparisons test comparing the mean change in weight between air and smoke exposure in ovariectomized (Ov) and non-ovariectomized mice was performed. No statistical significance was achieved at any of the time points. (TIF) [file pone.0164835.s003.tif]
